# MOET: a web-based gene set enrichment tool at the Rat Genome Database for multiontology and multispecies analyses

**DOI:** 10.1093/genetics/iyac005

**Published:** 2022-01-28

**Authors:** Mahima Vedi, Harika S Nalabolu, Chien-Wei Lin, Matthew J Hoffman, Jennifer R Smith, Kent Brodie, Jeffrey L De Pons, Wendy M Demos, Adam C Gibson, G Thomas Hayman, Morgan L Hill, Mary L Kaldunski, Logan Lamers, Stanley J F Laulederkind, Ketaki Thorat, Jyothi Thota, Monika Tutaj, Marek A Tutaj, Shur-Jen Wang, Stacy Zacher, Melinda R Dwinell, Anne E Kwitek

**Affiliations:** 1 Department of Biomedical Engineering, Medical College of Wisconsin, Milwaukee, WI 53226, USA; 2 Division of Biostatistics, Medical College of Wisconsin, Milwaukee, WI 53226, USA; 3 Department of Physiology, Medical College of Wisconsin, Milwaukee, WI 53226, USA; 4 Clinical and Translational Science Institute, Medical College of Wisconsin, Milwaukee, WI 53226, USA; 5 Information Services, Medical College of Wisconsin, Milwaukee, WI 53226, USA

**Keywords:** model organism database, gene set enrichment, rat, web tool, ontology, multiple species analysis, multiple ontology

## Abstract

Biological interpretation of a large amount of gene or protein data is complex. Ontology analysis tools are imperative in finding functional similarities through overrepresentation or enrichment of terms associated with the input gene or protein lists. However, most tools are limited by their ability to do ontology-specific and species-limited analyses. Furthermore, some enrichment tools are not updated frequently with recent information from databases, thus giving users inaccurate, outdated or uninformative data. Here, we present MOET or the Multi-Ontology Enrichment Tool (v.1 released in April 2019 and v.2 released in May 2021), an ontology analysis tool leveraging data that the Rat Genome Database (RGD) integrated from in-house expert curation and external databases including the National Center for Biotechnology Information (NCBI), Mouse Genome Informatics (MGI), The Kyoto Encyclopedia of Genes and Genomes (KEGG), The Gene Ontology Resource, UniProt-GOA, and others. Given a gene or protein list, MOET analysis identifies significantly overrepresented ontology terms using a hypergeometric test and provides nominal and Bonferroni corrected *P*-values and odds ratios for the overrepresented terms. The results are shown as a downloadable list of terms with and without Bonferroni correction, and a graph of the *P*-values and number of annotated genes for each term in the list. MOET can be accessed freely from https://rgd.mcw.edu/rgdweb/enrichment/start.html.

## Introduction

In recent years, functional genomics and transcriptomic data have increased exponentially due to the development of high-throughput technologies, next-generation sequencing in particular (Ghandikota *et al.* 2018; Hinderer *et al.* 2019; Raudvere *et al.* 2019). Thus, database resources are essential to make this information accessible, as are algorithms that facilitate interpretation of large datasets for generating novel hypotheses for functional validation. The Gene Ontology (GO) Consortium was developed as a resource to describe and curate functional similarities for such data to make meaningful interpretations and to computationally represent the current knowledge (Ashburner *et al.* 2000; The Gene Ontology Consortium 2019, 2021). GO creates a standardized vocabulary to define the biological processes, cellular components, and molecular functions associated with the gene. Currently, GO provides over 7 million annotations across multiple organisms (Boyle *et al.* 2004; Eden *et al.* 2009; The Gene Ontology Consortium 2021). These GO terms can be used for gene set enrichment analysis, which is a widely used process to statistically identify terms that are significantly overrepresented or enriched within a list of input genes or proteins (Pomaznoy *et al.* 2018; Zuniga-Leon *et al.* 2018). This has led to the development of many web-based applications and programs providing easy access for researchers from diverse scientific disciplines for a variety of uses (Khatri *et al.* 2004; The Gene Ontology Consortium 2017).

In the past years, numerous ontology analysis tools (Supplementary Table S1) have been developed (Berriz *et al.* 2003; Subramanian *et al.* 2005; Mi *et al.* 2021). Many of these tools are web-based, public, and free to use; others require licensing (e.g. IPA) (Kramer *et al.* 2014) or downloading a program (program-based) for their use (e.g. GSEA, BinGO) (Maere *et al.* 2005; Subramanian *et al.* 2005). A few ontology analysis tools require knowledge of specific programming languages, such as R (e.g. DOSE, GSEA) (Maere *et al.* 2005; Yu *et al.* 2015) or Python (e.g. GOATools) (Klopfenstein *et al.* 2018), or have operating system-specific requirements (e.g. FunRich), making them inconvenient for some users (Benito-Martin and Peinado 2015; Fonseka *et al.* 2021). Therefore, web-based tools, such as AmiGO, FunSet, and PANTHER are comparatively popular because of their ease to use (Carbon *et al.* 2009; Hale *et al.* 2019; Mi *et al.* 2021). However, most gene enrichment or overrepresentation tools are limited only to the analysis of specific species, and/or ontologies [e.g. WormBase enrichment analysis, Comparative GO (Fruzangohar *et al.* 2013; Angeles-Albores *et al.* 2018; Le 2020)].

Most of the gene enrichment or overrepresentation tools adopt a common strategy of entering a set of genes which are then compared statistically against a given background gene set (which may or may not be defined by the user) (Huang da *et al.* 2009a). Some of the tools display the results of the analysis in directed acyclic graphs (DAG), such as WEGO, GO:Term Finder and WebGestalt (Boyle *et al.* 2004; Ye *et al.* 2018; Liao *et al.* 2019). Of note, the development of ontologies is an ongoing process with terms being created, obsoleted, or merged regularly. Over the last 2 years, within GO, the number of terms created, obsoleted, and merged were 672, 661, and 752 terms, respectively (http://geneontology.org/stats.html; The Gene Ontology Consortium 2021). Several tools that determine ontology term enrichment are limited by a lack of frequent updates resulting in inaccurate outputs; for example, DAVID, FuncAssociate and WebGestalt were last updated in 2016, 2018, and 2019, respectively (Berriz *et al.* 2003; Jiao *et al.* 2012; Liao *et al.* 2019). Also, a few tools do not provide the user with multiple testing corrections for their analysis (e.g. Algal Functional Annotation Tool, Comparative GO) which is an important parameter in functional ontological evaluations due to the large volume of data (Lopez *et al.* 2011; Fruzangohar *et al.* 2013).

The Rat Genome Database (RGD) was developed in 1999 as a one-stop rat genomic and physiologic data repository. RGD has progressed to store information for mouse, human, chinchilla, bonobo, dog, pig, naked mole-rat, vervet (or green monkey) and 13-lined ground squirrel along with advanced RGD tools (Laulederkind et al. 2019; Smith *et al.* 2020). A major strength of RGD is its expert manual curation of genes demonstrated by 251,278 cumulative manual annotations. The analysis and visualization of all these data are facilitated by RGD tools such as advanced search tool OLGA (Object List Generator and Analyzer), InterViewer (protein-protein interaction viewer), and GOLF (Gene-Ortholog Location Finder) (Smith *et al.* 2020), with each of them providing unique features. In addition to manual curation efforts, RGD also imports data from other databases to provide the user with further information for their analysis. To improve on gaps in the term enrichment field and to meet the growing complex experimental needs of the research community, RGD has developed the Multi Ontology Enrichment Tool (Supplementary Table S1) (MOET, https://rgd.mcw.edu/rgdweb/enrichment/start.html). MOET is a unique web-based ontology analysis tool that generates a list of terms statistically overrepresented within the user's genes of interest, leveraging multiple classes of ontology annotations. Some of the advantages of MOET over the currently available enrichment tools are:

- It is a web-based, publicly available, and freely accessible ontology analysis and visualization tool.
- It is simple to use, and results are provided with a few clicks in seconds; no software installations or programming skills are required.
- It provides functionality for multiple ontologies, including Disease, GO, Pathway, Phenotype, and Chemical entities (ChEBI).
- It provides enrichment analysis for multiple RGD species, including rat, mouse, human, bonobo, squirrel, dog, pig, chinchilla, naked mole-rat and vervet (green monkey).
- The *P*-values are displayed for each term in the output with Bonferroni multiple testing corrections to control false positives.
- It supports input of any of 11 different common identifier types, saving the user from translating one type of ID to an acceptable input ID.
- It is updated weekly, providing the user with the most recent data for analyses.

## Methods

### Data that support the tool

**Fig. 1.**
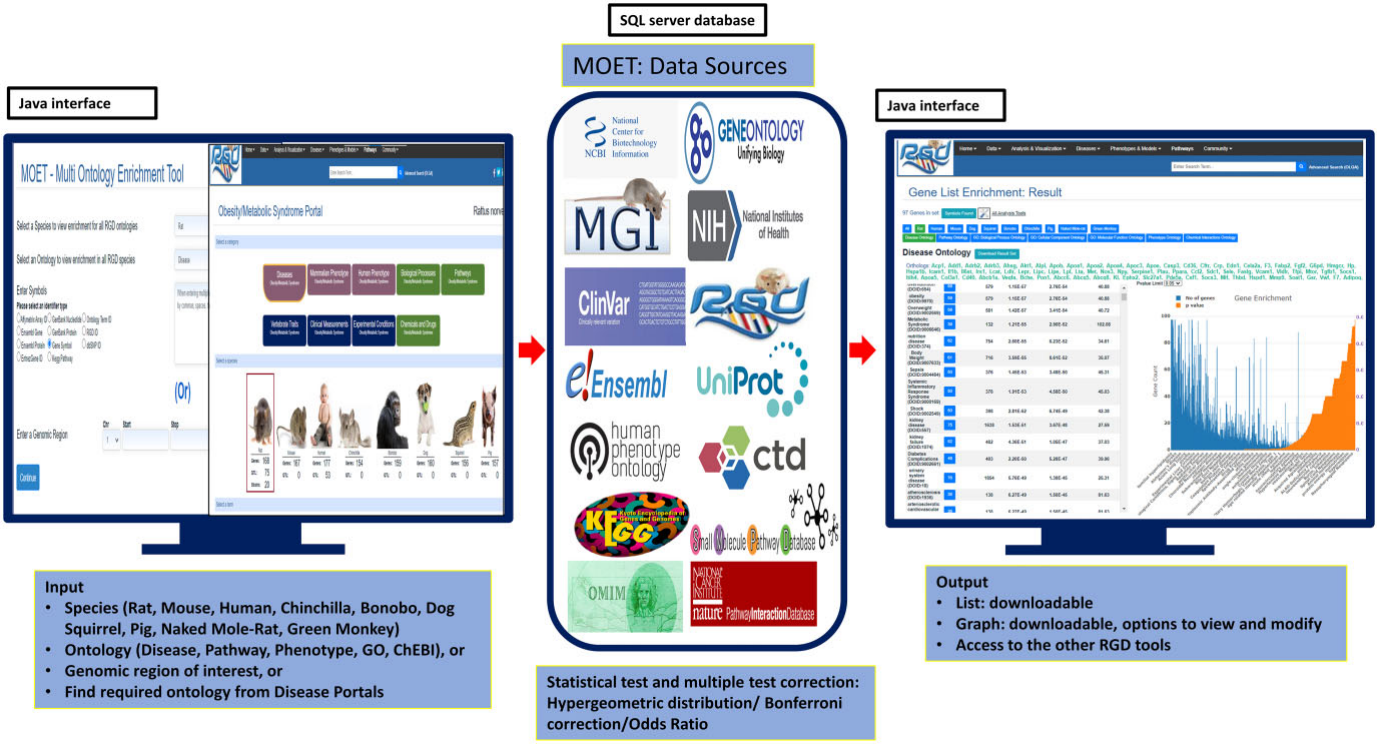
Schematic overview of MOET functionality. The user provides input using the options shown, MOET uses data integrated at RGD from all the sources, performs enrichment and statistical analysis, and provides downloadable results.

The backend database for MOET consists of RGD's extensive corpus of functional annotations derived from manual curation at RGD, supplemented with automated pipelines that import and integrate data from multiple databases (Fig. 1). The curators at RGD use in-house designed curation software integrated with an RGD-developed literature search tool OntoMate (Liu *et al.* 2015) to identify peer-reviewed journal articles related to a specific disease category and create annotations based on genes for RGD species, in addition to annotations imported from other data sources. Disease, pathway, and ChEBI annotations for rat, mouse, and human studies are annotated at RGD with appropriate evidence codes for the data types. Annotations are assigned to orthologous genes in other species using the inferred from sequence orthology (ISO) evidence code. Rat-specific GO annotations and rat and human gene-specific phenotype annotations are curated manually from the same gene list at RGD. To provide integrated disease-focused environments, RGD has developed 15 Disease Portals (Shimoyama *et al.* 2016) (as of September 2021); the portals are designed to be entry points for disease-focused researchers to access integrated information related to their area of interest including disease-specific annotations, tools and datasets.

The data sources for imported ontology annotations for rat, mouse, and human include: UniProt’s Gene Ontology Annotation group (UniProt-GOA) for rat GO annotations from other groups, including rat Inferred from Electronic Annotation (IEA) annotations (UniProt Consortium 2021); Gene Ontology Resource for human and mouse GO annotations (The Gene Ontology Consortium 2021); Mouse Genome Informatics for Mammalian Phenotype (MP) Ontology annotations for mouse, and disease annotations for human and mouse (Baldarelli *et al.* 2021); Online Mendelian Inheritance in Man (OMIM) for human disease data (Amberger and Hamosh 2017); Online Mendelian Inheritance in Animals (OMIA) for disease and phenotype annotations for dog and pig (Nicholas 2021); ClinVar for human disease annotations (Landrum *et al.* 2018); Comparative Toxicogenomics Database (CTD) for rat, mouse and human gene-chemical interaction annotations and human disease annotations (Davis *et al.* 2019); Human phenotype ontology group for HPO annotations (Köhler *et al.* 2021); and Small Molecule Pathway Database (SMPDB) for human pathway annotations (Jewison *et al.* 2014). In addition to the regular import of current data, RGD has data archived from the Kyoto encyclopedia of genes and genomes (KEGG) for pathway annotations (Kanehisa *et al.* 2021) 8 years ago. Also, RGD stores data from retired databases, namely the Pathway Interaction Database (Schaefer *et al.* 2009), and the Genetic Association Database from which the last data downloads were in 2012 and 2014, respectively (Becker *et al.* 2004).

### Software development and availability

MOET is built on J2EE technologies (http://java.sun.com/j2ee/overview.html) and driven off the RGD Oracle database. Backend technologies include the popular Spring framework and Swagger REST API. The user interface is built on the Vue.js JavaScript framework, Bootstrap front-end toolkit, and Plotly charting library. The hypergeometric distribution for the statistical hypothesis testing is created, and *P*-values are calculated, using the Apache commons library. Supported browsers include Microsoft Edge, Mozilla Firefox, Google Chrome and Apple Safari. MOET source code and documentation are available from Github at https://github.com/rat-genome-database/rgd-web-application/tree/master/web-app/WEB-INF/jsp/enrichment. An example file (example.jsp) has been included as well for users to run their instance using the source code. In addition, RGD provides public APIs to perform MOET analyses (https://rest.rgd.mcw.edu/rgdws/swagger-ui.html#/enrichment-web-service). The source code is reusable and can be used to set up an instance based on the RGD application.

### Calculation of *P*-values and multiple testing corrections

Various statistical approaches to calculate the *P*-value are used in different ontology tools. Fisher’s exact test and the hypergeometric test are thought to be more precise than other tests and are used commonly in such analyses (Huang da *et al.* 2009a). MOET’s algorithm is also based on the hypergeometric test.

Since multiple tests (the number of terms associated with genes in the reference list) are run in parallel, MOET performs the Bonferroni correction to control the type I error (false positive) (Farcomeni 2008). The results list also provides the odds ratio (https://rgd.mcw.edu/wg/new-moet-algorithm/) for each term to quantify the strength of the enrichment between input gene list and each term. It is defined as the ratio of the odds of occurrence for an ontology term in the input list and the odds of occurrence for an ontology term in the reference set.

### The user interface

**Fig. 2.**
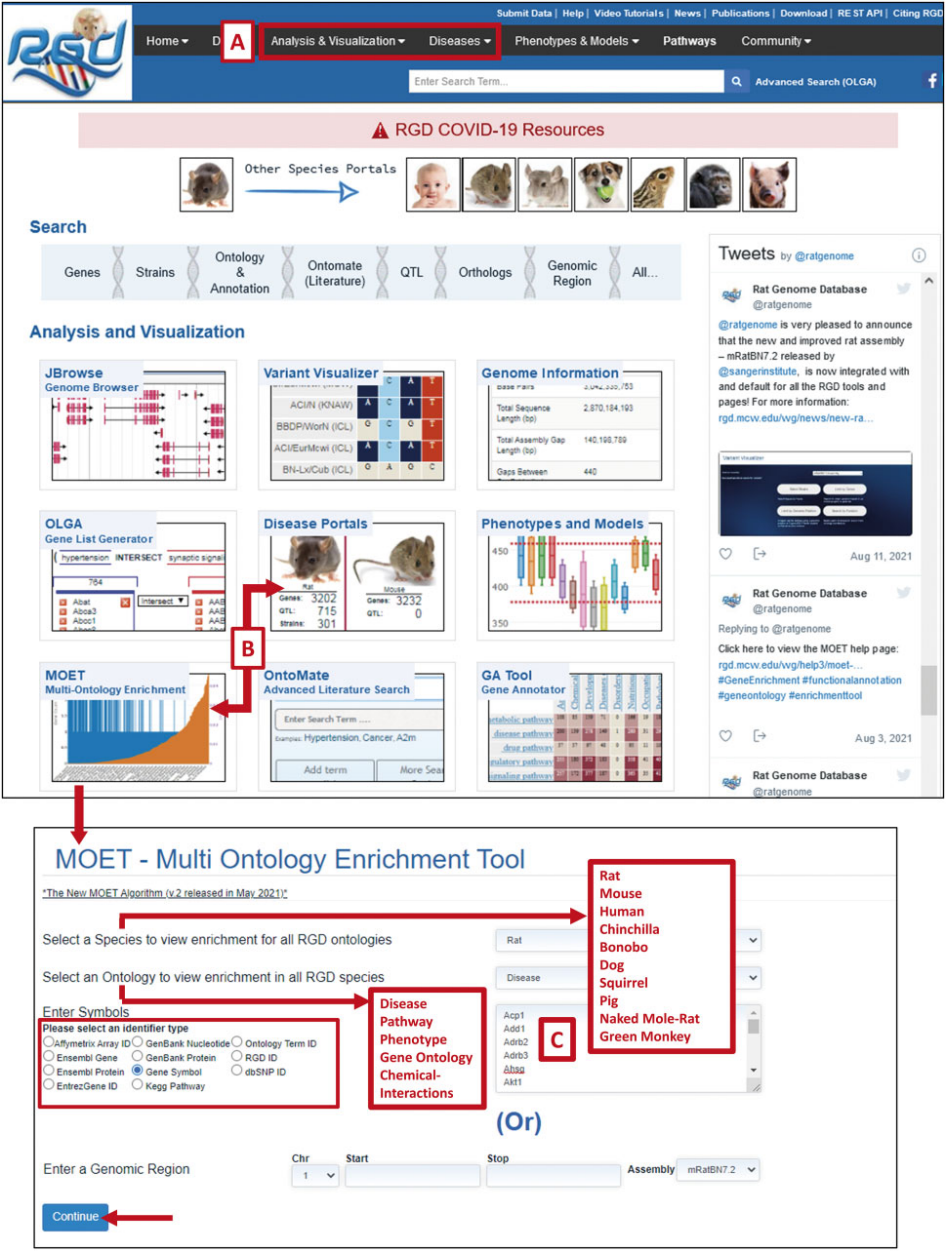
Access to MOET from A) and B) “Analysis & Visualization” and individual disease pages from “Diseases” and “Disease Portals”; C) You can enter your gene or protein list in the MOET interface as one of the acceptable RGD identifier types and then click on continue to view the results.

MOET is accessible from the RGD front page from “Analysis & Visualization” in the toolbar menu or as one of the tools embedded into RGD's individual Disease Portal pages from “Diseases” and “Disease Portals” (Fig. 2, A and B). MOET’s front page allows the use of an input list of genes or proteins in numerous identifier types with the desired species and ontology selection (Fig. 2C, Supplementary Fig. S1A1–A3). Alternatively, a genomic region of interest with the applicable assembly can be entered (Supplementary Fig. S1A4).

### Visualization of output and downstream analyses

**Fig. 3.**
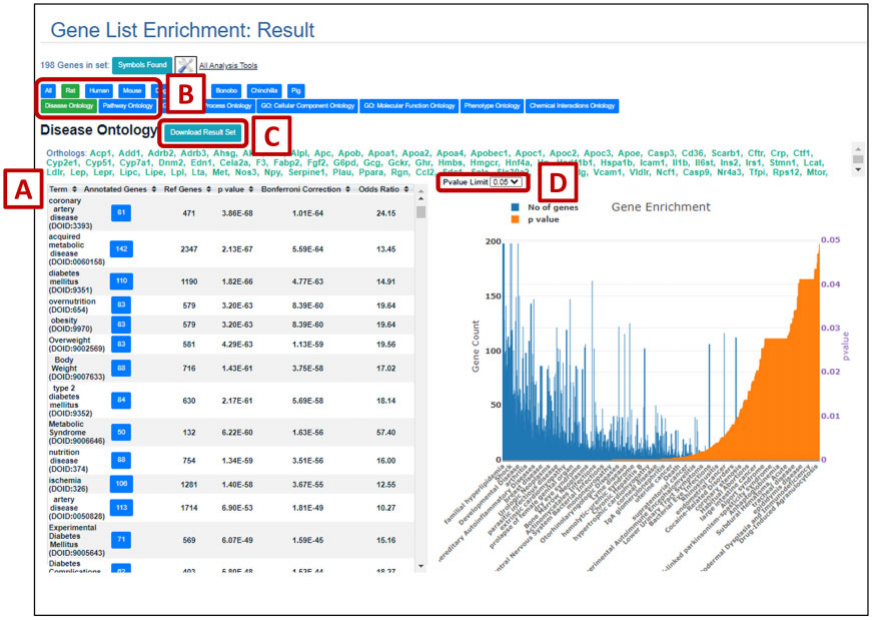
MOET results page with downloadable list and graph; A) A list of over-represented terms with the uncorrected and Bonferroni-corrected *P*-values and odds ratio for each term; B) The species or ontology used for the analysis can be changed with the click of a single button. Current display is for rat and disease as indicated by the green tabs. Click Human and Pathway Ontology to obtain over-represented pathway terms for the list of human orthologs of the original input list of rat genes (refer to Supplementary Fig. S1); C) The result list can be downloaded. The resulting file contains the list of terms with the counts of genes annotated to each term in the input set and the reference set, the *P*-value, Bonferroni correction and odds ratio. D) The results shown in the graph of the number of annotated genes and *P*-value for each term in the result set can be made more or less stringent by changing the *P*-value limit using the drop-down list of options.

The results are displayed on the results page as a downloadable list (Fig. 3, A–C, Supplementary Fig. S1. B and C) and a graph. The functionality to perform comparisons across species and ontologies is provided by clicking on the species or ontology from the top of the results page (Supplementary Fig. 1C). Also, the *P*-value limit can be adjusted (Fig. 3D) with the available choices (0.01, 0.05, and 0.1) to modify only the displayed graph results accordingly (Supplementary Fig. S1C3A). The graph can be explored using the self-explanatory buttons at the top of the graph. Further, the user can send the list of genes to the other RGD analysis tools with the “All Analysis Tools” button and the resulting toolbox options (Supplementary Fig, S1B3).

The number of annotated genes (Supplementary Fig. S1B2) beside the term in the table opens a pop-up containing the list of genes annotated to that term that can in turn be explored with MOET using the “Explore this Gene Set” link. The “All Analysis Tools” button and the toolbox can be used here to further analyze these genes with the other RGD tools.

**Fig. 4.**
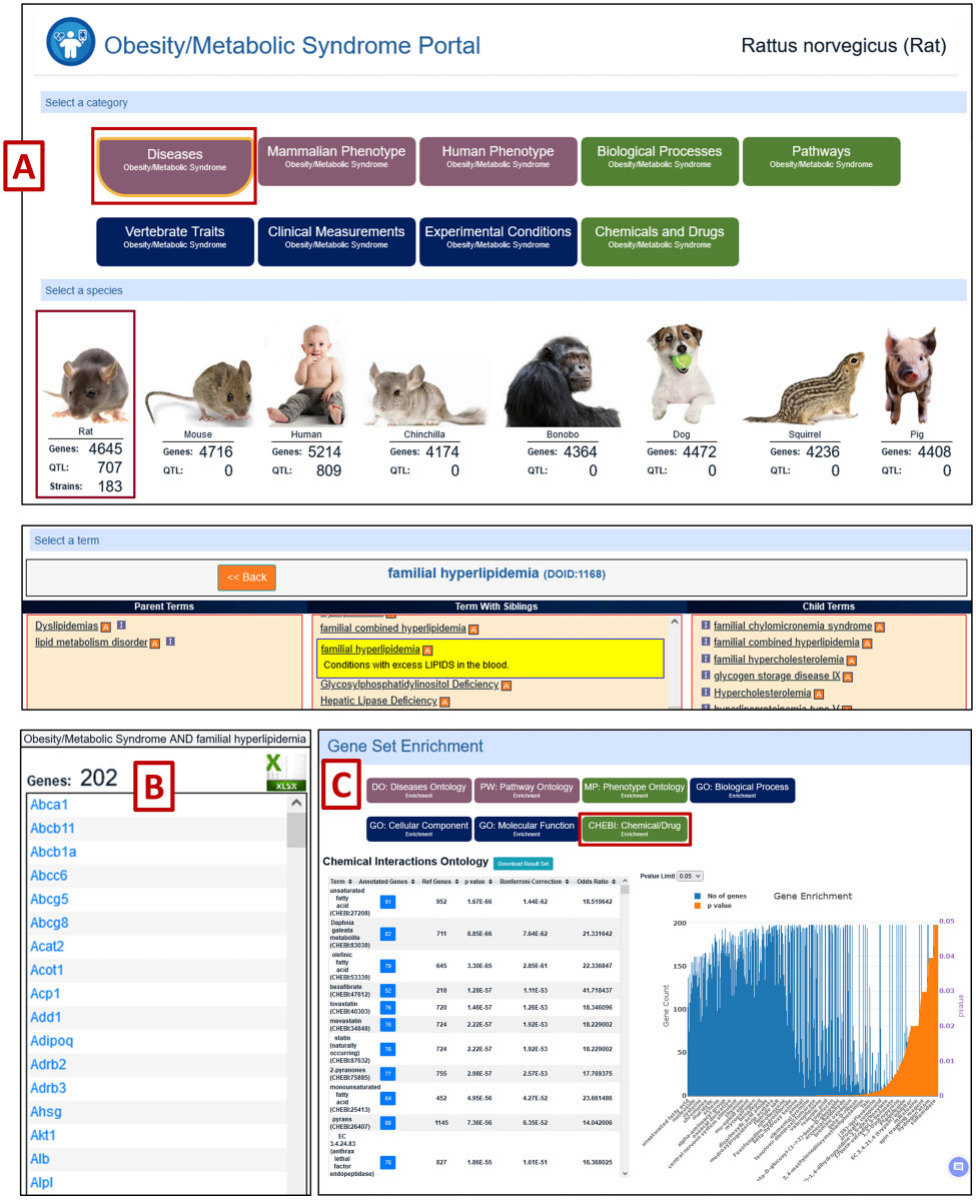
MOET is accessible from individual Disease Portal pages. Here “Obesity/Metabolic Syndrome Portal” is shown; A) Rat as species and Disease as Ontology Category are selected as default; B) Number of genes associated with the selected species and disease category annotated to the term “familial hyperlipidemia” are shown; C) You can interchangeably select a different ontology for MOET analysis from the buttons below “Gene Set Enrichment.” Here, ontology analysis results for Rat in Chemicals and Drugs or ChEBI ontology are shown with “unsaturated fatty acid” as the top term.

Additional means to reach MOET are from the individual Disease Portals from “Diseases” in the toolbar at the top or “Disease Portals” in the RGD front page panel menu (Fig. 2, A and B). The user can select the required species and ontology category (Supplementary Fig. S2A) from the individual Disease Portal page (Kaldunski *et al.* 2021). Each Disease Portal has the associated gene, strain and QTL data integrated with it (Supplementary Fig. S2B). The “Gene Set Enrichment” section at the bottom of the disease page is integrated with MOET and sends the list of genes to MOET for analysis (Fig. 4, Supplementary Fig. S2C). Other ontologies can be selected from the bottom of the page below “Gene Set Enrichment” to analyze the list of genes annotated to the selected term for a different ontology.

### Software comparison

**Fig. 5.**
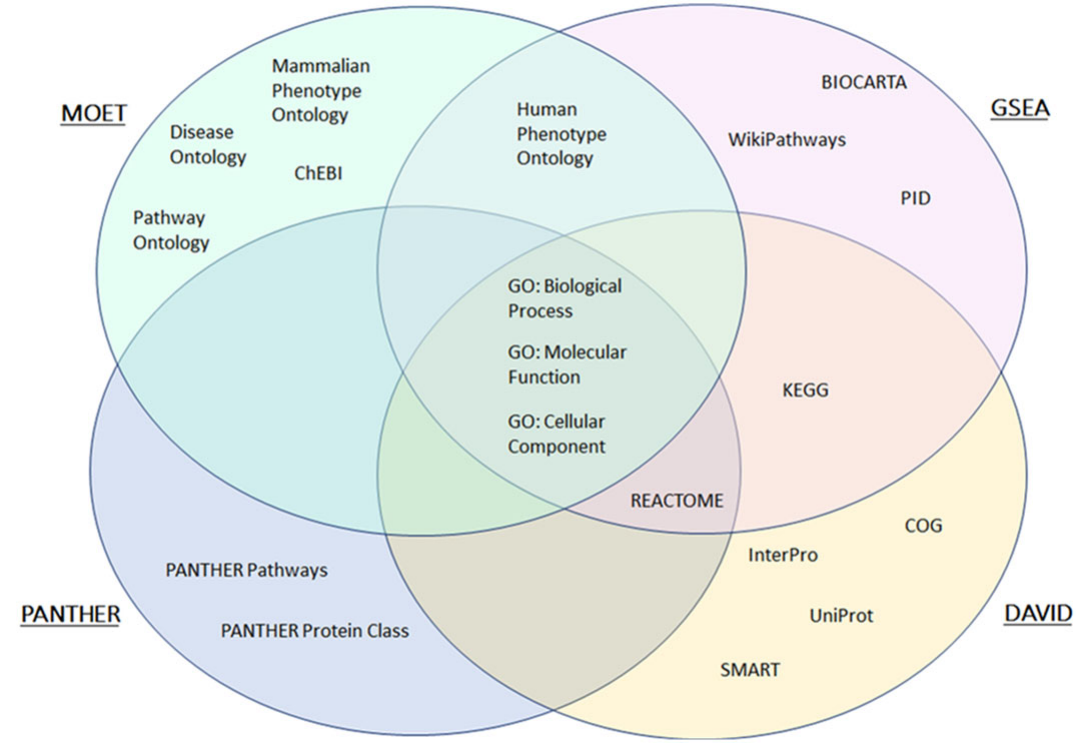
Intersecting curated canonical pathways and ontologies used in software comparison. This Venn diagram depicts common and unique resources used by MOET, PANTHER, GSEA, and DAVID for integration into their respective ontology and pathway analyses. Several resources including KEGG and UniProt are included in the development of MOET ontologies, but results to their specific terms are not provided from the analysis.

To demonstrate the utility of MOET results a use-case was constructed from an experimentally derived gene set. Several popular enrichment tools were also selected to analyze these data and generate a results comparison. The software programs used in the comparison were MOET, DAVID (Huang da *et al.* 2009b), GSEA (Subramanian *et al.* 2005), and PANTHER (Mi *et al.* 2021). To generate a comparison that was consistent across the programs, several limiting factors were selected to maintain uniformity. An experimental gene set (Wang *et al.* 2015; GSE50027) of differentially expressed genes (DEG) from RNAseq analysis of liver samples from Lyon Hypertensive (LH/MavRrrcAek; RRID: RGD_10755352) and Lyon Normotensive (LN/MavRrrcAek; RRID: RGD_10755354) rats was used as a test gene set. These animals serve as a control (LN) and a disease (LH) model exhibiting many characteristic phenotypes associated with metabolic syndrome including hypertension, obesity, and dyslipidemia (Dupont *et al.* 1973; Vincent *et al.* 1993). The unfiltered DEG set began with 630 genes. The gene set that was submitted to each program was represented by Ensembl gene identifiers from the original published data (Wang *et al.* 2015). A common, minimal gene set of 411 genes recognized by all software was generated and used in the subsequent analyses (Supplementary Table S2). To maintain consistency, rat was selected as the species for genetic reference in all programs, and analyses were limited to intersecting curated canonical pathways and ontologies between the tools (Fig. 5). While each software included a pathway analysis, the resource data differed among them. As a result, limiting vocabularies to those common amongst all programs left only GO sets (Biological Process, Molecular Function, and Cellular Component) for inclusion in the analyses (Fig. 5).

### Enrichment comparison between curated gene set and experimental gene set

To further contextualize the enrichment results obtained from MOET analysis of the experimental gene set, a comparison was conducted with a curated metabolic disease gene set from the disease ontology (DO) (DOID:0060158, acquired metabolic disease). For this comparison, a gene set annotated to DOID:0060158 was downloaded from RGD’s Obesity & Metabolic Syndrome Disease Portal (Supplementary Table S3) and was compared against an experimental gene set of LN vs LH liver DEG genes. The MOET-recognized experimental gene set from the LN *vs.* LH liver samples included 551 genes while the DO-acquired metabolic disease gene set contained 2,222 genes. Between these gene sets, there were 110 common genes. Each gene set was loaded in MOET and analyzed within each of MOET’s ontologies (GO, DO, PW, MP, and ChEBI) using rat as the reference species. The results within each ontology were compared between gene sets to determine common enriched annotated terms. The first comparison included all terms with Bonferroni-corrected *P*-values less than 0.05. The common genes between the gene sets were then loaded into Variant Visualizer at RGD for genomic assessment (Shimoyama *et al.* 2015). The LN and LH rat strain genomes were selected for comparison to the mRatBN7.2 reference sequence. In the assessment, single nucleotide variants (SNVs) residing within genes were included if the read depth was greater than 15x and nucleotide calls were homogenous.

## Results

### Functional characterization of MOET

To demonstrate the functionality of MOET, a use case was designed using an experimentally derived gene set. The MOET results are compared with results from several popular tools to determine commonality with the established tools in the identification of functional terms relevant to the gene set. The follow-up assessment provides a potential workflow that a MOET user may follow to add functional context to their gene set. Results from the tool were also used in conjunction with RGD’s resources to identify novel paths for future exploration.

### Software analysis comparison

**Fig. 6.**
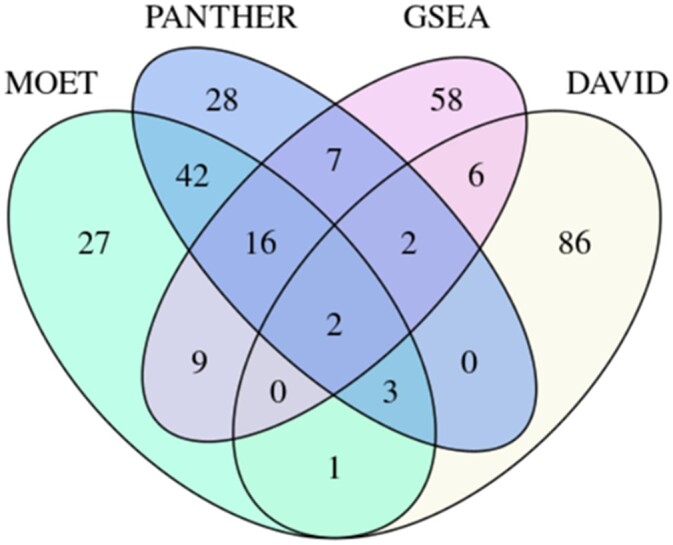
Representation of top 100 GO term overlap among compared enrichment tools. Gene Ontology analysis was performed using an experimentally derived DEG set. Genes were loaded into each software (MOET, PANTHER, GSEA, and DAVID). The top 100 terms ranked on *P*-value were assessed and overlaps are depicted in the Venn diagram. MOET has 63 terms in common with PANTHER, 27 terms with GSEA, and 6 terms with DAVID.

**Fig. 7.**
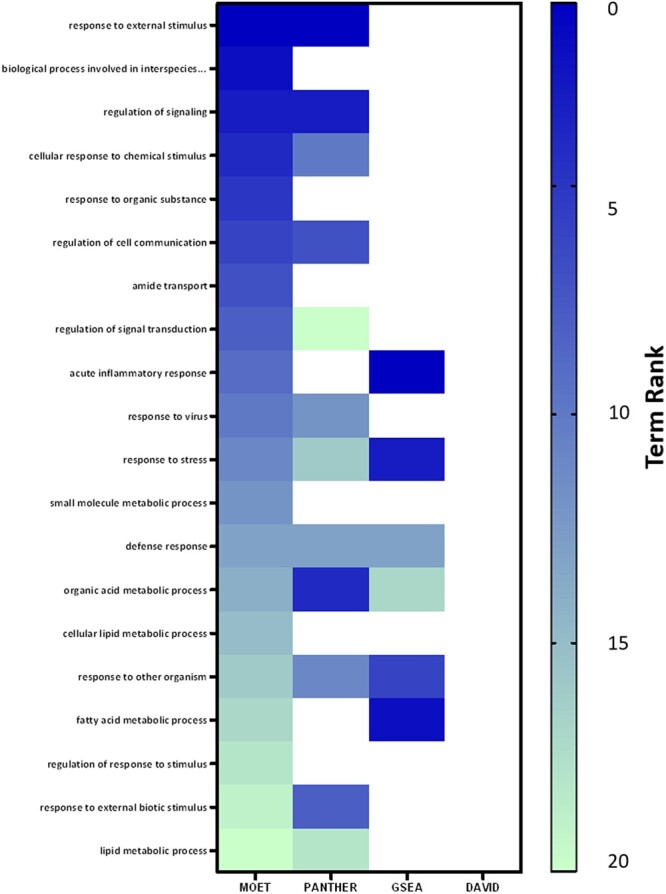
MOET top 20 GO biological process term overlap with compared enrichment tools. MOET contained 6 additional terms from the top 20 that were found in the top 100 terms from results in comparison software.

The experimental common minimal gene set of 411 genes (Supplementary Table S2), differentially expressed between liver RNA from LH and LN rats, was used for this analysis to demonstrate a use case for MOET while comparing it with common ontology analysis tools, including PANTHER, GSEA and DAVID. The top 100 GO terms listed by ascending multiple testing corrected *P*-values were included in the results from each program. The resulting terms were compared and were included in Supplementary Table S4 if the term occurred in MOET and at least 1 other program’s top 100 list (not necessarily significantly enriched terms). The table displays the term name, GO ID, ontology name, software, and multiple testing corrected *P*-values. The list is ordered first by the number of programs containing the term, and then sorted by MOET *P*-values. Figure 6 gives an overview of the term overlap between software analyses. The MOET analysis results overlap with at least one other tool in 73 of its top 100 terms. Its greatest correspondence between tested software was with the PANTHER analysis tool (63 terms) followed by GSEA (27 terms) and DAVID (6 terms) (Fig. 6). There were 2 terms in common among all 4 programs (endoplasmic reticulum, GO:0005783; innate immune response, GO:0045087), 19 terms in common among 3 programs, and 52 terms in common between any of the 2 programs (Supplementary Fig. S3 and Table S4). Following the 2 terms in common between all programs, 5 of the next 10 terms have a high representation of terms associated with metabolic function (Supplementary Fig. S3 and Table S4). The cumulative nonoverlapping terms from the top 100 lists of each program represented 199 terms. Of these terms, 78 had multiple testing corrected *P*-values below 0.05 with 71 of them having corrected *P*-values below 0.01 (Supplementary Table S5). Terms unique to the MOET analysis accounted for 27 of the 199 terms and 7 of these terms had corrected *P*-values less than 0.05 (Fig. 6, Supplementary Table S5). An additional assessment on the top 20 GO Biological Process specific terms from MOET analysis had ten overlapping terms that occurred in the top 20 list of the other programs (Supplementary Table S6 and Fig. 7). Our results showed that MOET results are generally comparable and consistent with the commonly available ontology analysis tools. However, each tool (including MOET) also has differences that set them apart from one another. These differences could be attributed to some of the exclusive MOET features, and dissimilarities in statistical formulae, multiple testing corrections, ontologies, species, gene identifiers, and frequency of data updates between the tools. We have described the factors contributing to the differences in the Discussion section of this manuscript.

### Enrichment comparison between curated gene set and experimental gene set

The overlapping terms from each gene set analysis (experimental gene set and DO-acquired metabolic disease gene set) within each ontology generated a list of 103 common terms from non-ChEBI ontologies and 1,829 terms from ChEBI (Supplementary Tables S7 and S8). To refine the list of common terms, a comparison between the top 25 ranked term lists within each ontology was performed. This assessment produced a list of 47 common terms: 5 from DO, 3 from PW, 4 from GO, 7 from MP, 3 from HPO, and 25 from ChEBI (Supplementary Tables S7 and S9). Terms in common from these results in DO included diabetes mellitus (DOID:9351) and glucose metabolism disease (DOID:4194; Supplementary Tables S8 and S9). Seven of the overlapping terms originating from PW and GO supported an association of the gene sets with a metabolic function.

Variant visualizer identified 4,971 SNVs that differed between LN and LH in the common gene set from the 110 overlapping genes. Several of these SNVs led to amino acid (AA) changes with PolyPhen-predicted possible or probable damage to the resulting protein (Supplementary Tables S10 and S11). One gene had a predicted damaging SNV unique to LN (*Slc11a1*), and 1 gene had 2 predicted damaging SNVs unique to LH (*Enpp1*) (Supplementary Tables S10 and S11). SNVs within *ENPP1* are associated with traits relevant to metabolic syndrome (blood phosphate measurement and c-reactive protein measurement) (Buniello *et al.* 2019), cardiovascular diseases (Bacci *et al.* 2011), obesity, increased risk of glucose intolerance and type 2 diabetes in humans (Meyre *et al.* 2005). Thus, integrating MOET with the other RGD tools led to identification of possible candidate genes for metabolic syndrome.

## Discussion

GO over-representation analysis is an effective way to facilitate the analysis and interpretation of large amounts of -omics data. MOET, developed at RGD, is an ontology analysis tool that implements its assessment utilizing a web-based application. It provides 6 different ontology analyses with all the RGD species in an intuitive and user-friendly manner aiming for ease of use for researchers, particularly those without an extensive computer science background. MOET doesn’t need installation or preparation of local databases for its use. MOET, therefore, can utilize its integrated resources to facilitate novel, functional interpretation of user gene sets.

This introduction to MOET has highlighted many of the distinctive features that establish it as an easily accessible tool that provides unique analysis across multiple ontologies and species. Characterization of the tool with other popular tools commonly used for enrichment analysis has demonstrated consistency amongst results when using a benchmark gene set while providing a unique pattern of enriched terms. The comparison provided in this work used a gene set derived from DEGs found between a rat strain representing a model of metabolic syndrome (LH) and a control strain (LN). MOET generated overlapping results with established currently available tools and produced annotated term results from the GO which support a metabolic role for the differentially expressed gene set. It can be concluded that there is a good overlap with significant terms between MOET and other ontology analysis tools.

In our comparison, in addition to the commonality between terms, we also found differences in the number of annotations and *P*-values between the tools used for comparison. One source of difference could be that DAVID, PANTHER, GSEA, and MOET are based on different algorithms and use different methods for multiple testing corrections (Supplementary Table S1). Differences in the number of annotations and *P*-values between these tools can be attributed to some of the benefits that are unique to MOET. The continuing updates in ontology and annotations cause differences in significance values as new parent-child relationships increase the number of annotations to a term. Since MOET draws its underlying data directly from the RGD database, which is updated on a weekly basis, it has the most up-to-date ontologies and annotations resulting in the most accurate significance values. Another unique feature of MOET is its algorithm that includes only the genes in the selected species annotated to the selected ontology as the reference set. Thus, the *P*-value calculation is more precise compared to other tools which consider the entire gene list (global reference, e.g. DAVID) or require a user input a reference list.

The purpose of developing MOET was to provide the research community with a means to interpret experimentally derived gene sets. The breadth and depth of any scientific discipline and any individual researcher inherently have gaps in understanding and experience. The ontologies and species chosen for MOET are specifically designed to generate coverage for these gaps and produce functionally interpretable results. The description of MOET’s operability along with the use case provided in the results above establish a potential workflow to enable functional characterization of user-generated gene sets. An indication of support from the research community can be seen through MOET's usage since its first public release in April 2019. The stand-alone tool has been directly accessed over 9,000 times (September 2021 Google analytics query) and this count is likely an under-representation since MOET is also embedded into the Disease Portals which are not included in Google analytics counts.

RGD continues its commitment to providing the best in data and software tools for the research community. Future updates in MOET will include support for enrichment analysis that incorporates expression results and implementation of additional algorithms for *P*-value calculation. We also plan to integrate the option of showing a negative correlation between the genes and their respective annotated terms. We value feedback from the research community and strive to incorporate input and comments from users that assist in our software navigation and functionality. Each page in RGD has a link to send feedback or feedback can be submitted in the “Contact Us” form (https://rgd.mcw.edu/contact/index.shtml) at RGD.

## Data availability

The authors state that all data necessary for confirming the conclusions presented in the article are represented fully within the article. MOET is freely available at https://rgd.mcw.edu/rgdweb/enrichment/start.html. MOET source code and documentation are available from Github at https://github.com/rat-genome-database/rgd-web-application/tree/master/web-app/WEB-INF/jsp/enrichment.

Supplemental material is available at *GENETICS* online.

## Supplementary Material

iyac005_Supplementary_Figure_1

iyac005_Supplementary_Figure_2

iyac005_Supplementary_Figure_3

iyac005_Supplementary_Tables_S1-S11
