# Supplementary material for: MOET: a web-based gene set enrichment tool at the Rat Genome Database for multiontology and multispecies analyses
Source: Genetics. 2022 Jan 28;220(4):iyac005. doi: 10.1093/genetics/iyac005 (PMC8982048; doi:10.1093/genetics/iyac005)
Supplement: iyac005_Supplementary_Figure_1 [file iyac005_supplementary_figure_1.pdf]

Supplementary Figure 1

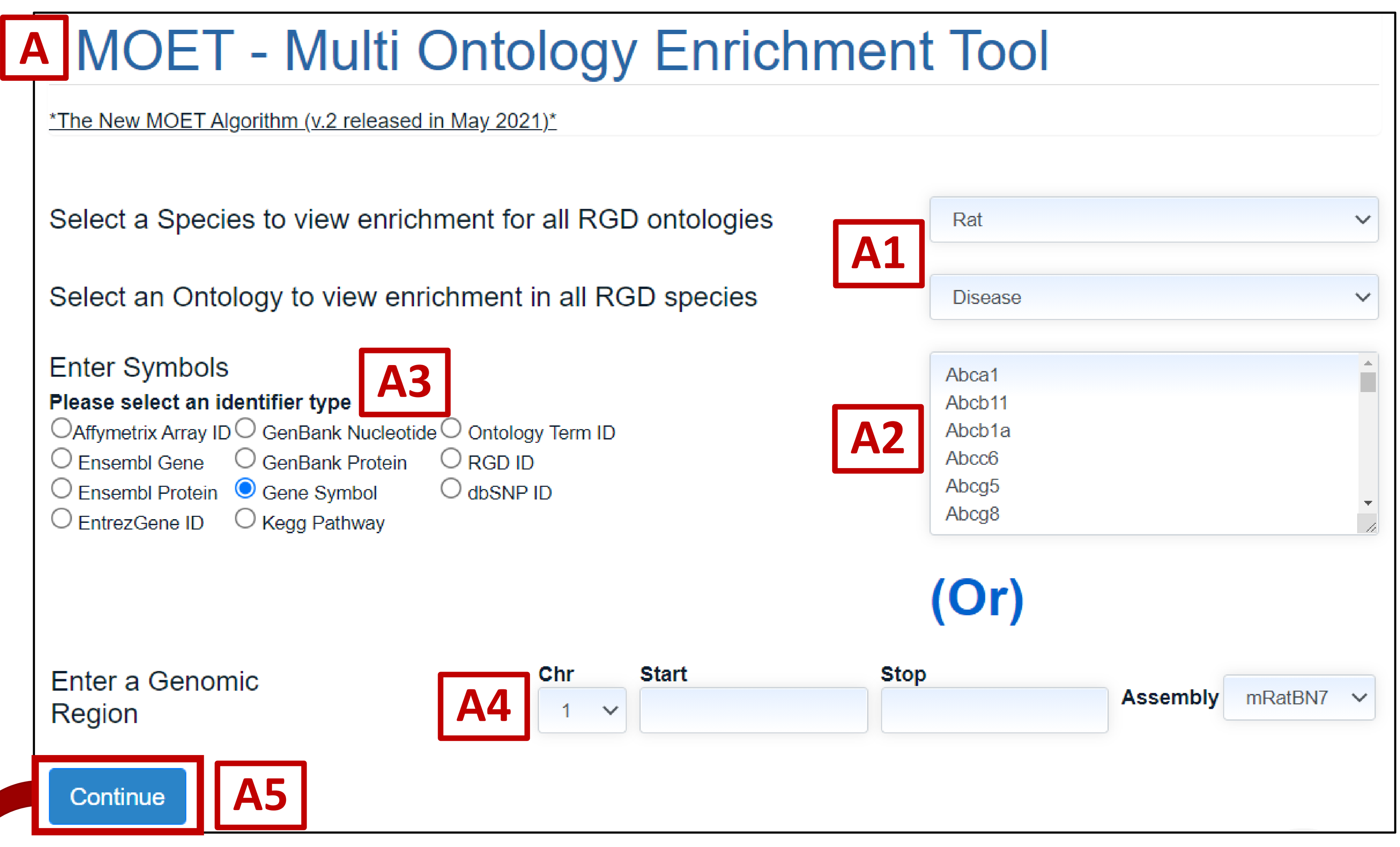

Figure S1A: MOET provides flexible options for beginning an analysis. Select from all of RGD's species (rat, human, mouse, dog, pig, chinchilla, squirrel, naked mole-rat, green monkey and bonobo) and/or ontologies (disease, phenotype, pathway, GO and ChEBI) (S1A1) and enter a list of identifiers (Affymetrix Array ID, GenBank Nucleotide, Ontology Term ID, Ensembl Gene ID, GenBank Protein ID, RGD ID, Ensembl Protein ID, Gene Symbol, dbSNP ID, EntrezGene ID, KEGG Pathway ID) (S1A2). The default is to enter gene symbols but any of the listed ID types are acceptable (S1A3). Alternatively, choose a sequence assembly for your selected species and enter the position of a genomic region (S1A4). Clicking 'Continue' (S1A5) initiates the enrichment analysis.

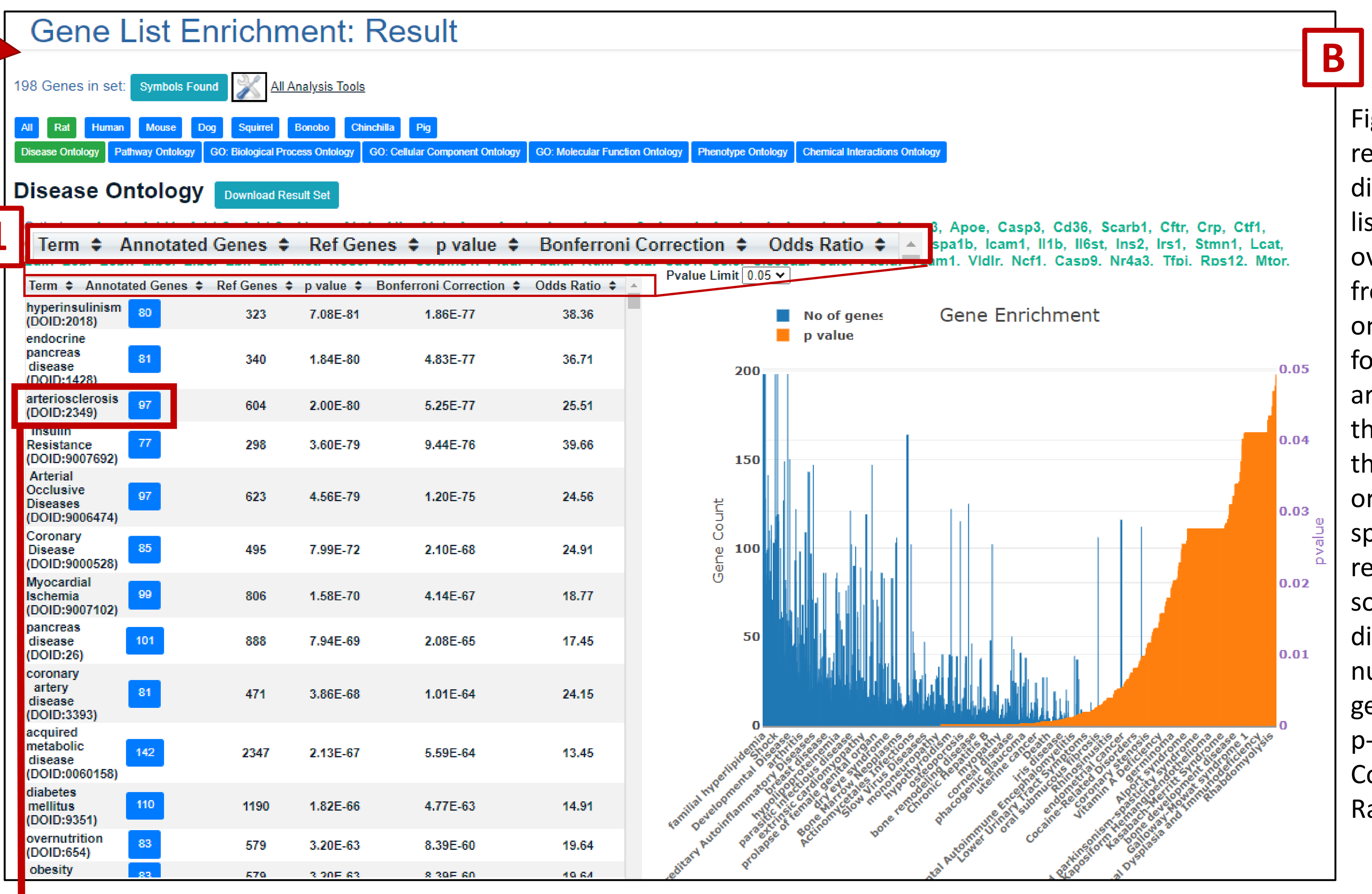

Figure S1B: The MOET result page. MOET displays the results as a list and a graph of overrepresented terms from all of the ontologies used by RGD for gene curation that are over-represented in the annotations for those genes, or orthologs in other species. (S1B1) The results in the list are sortable and are displayed with the number of Annotated genes, reference genes, p-value, Bonferroni Correction and Odds Ratio for each term.

(S1B2) Click on the button that shows the number of annotated genes to see the list of genes annotated to that term. The gene list can be sent again to MOET for analysis using the 'Explore this Gene Set' option. The 97 genes annotated to 'arteriosclerosis' are selected here.

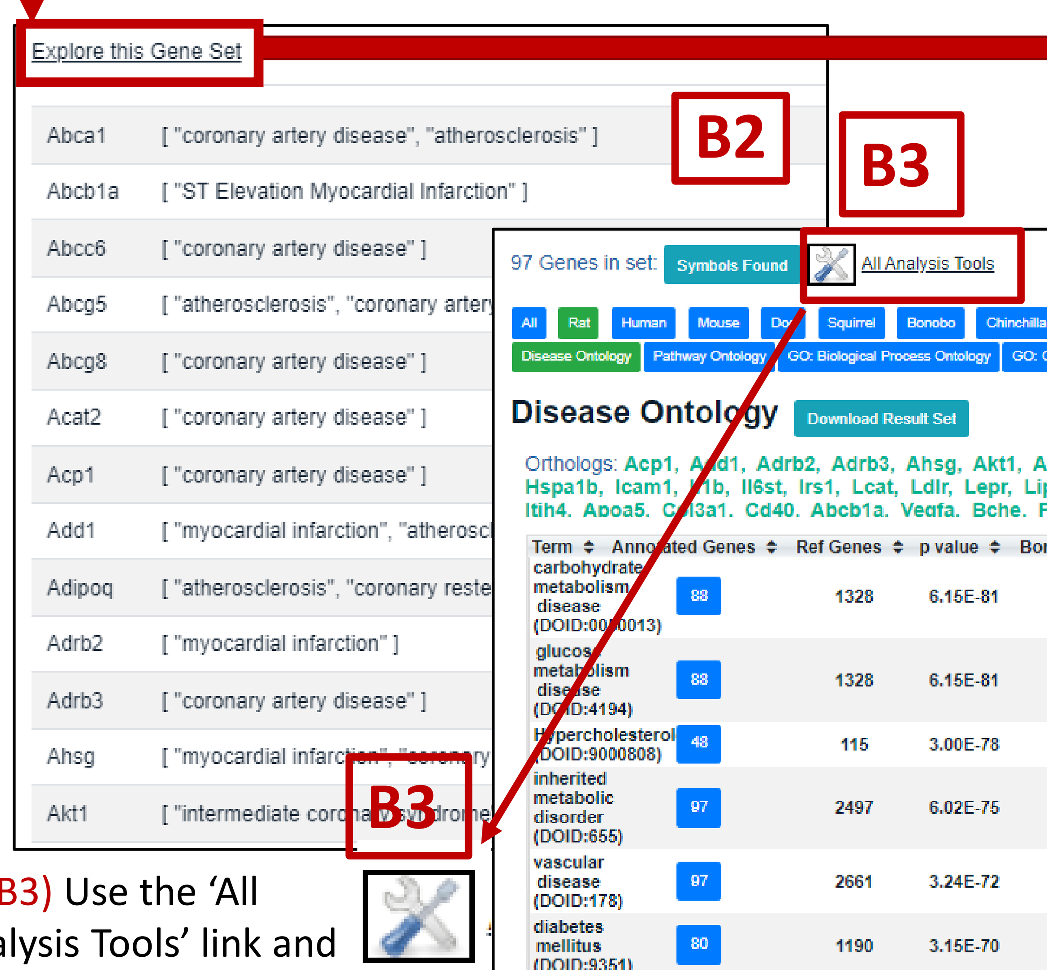

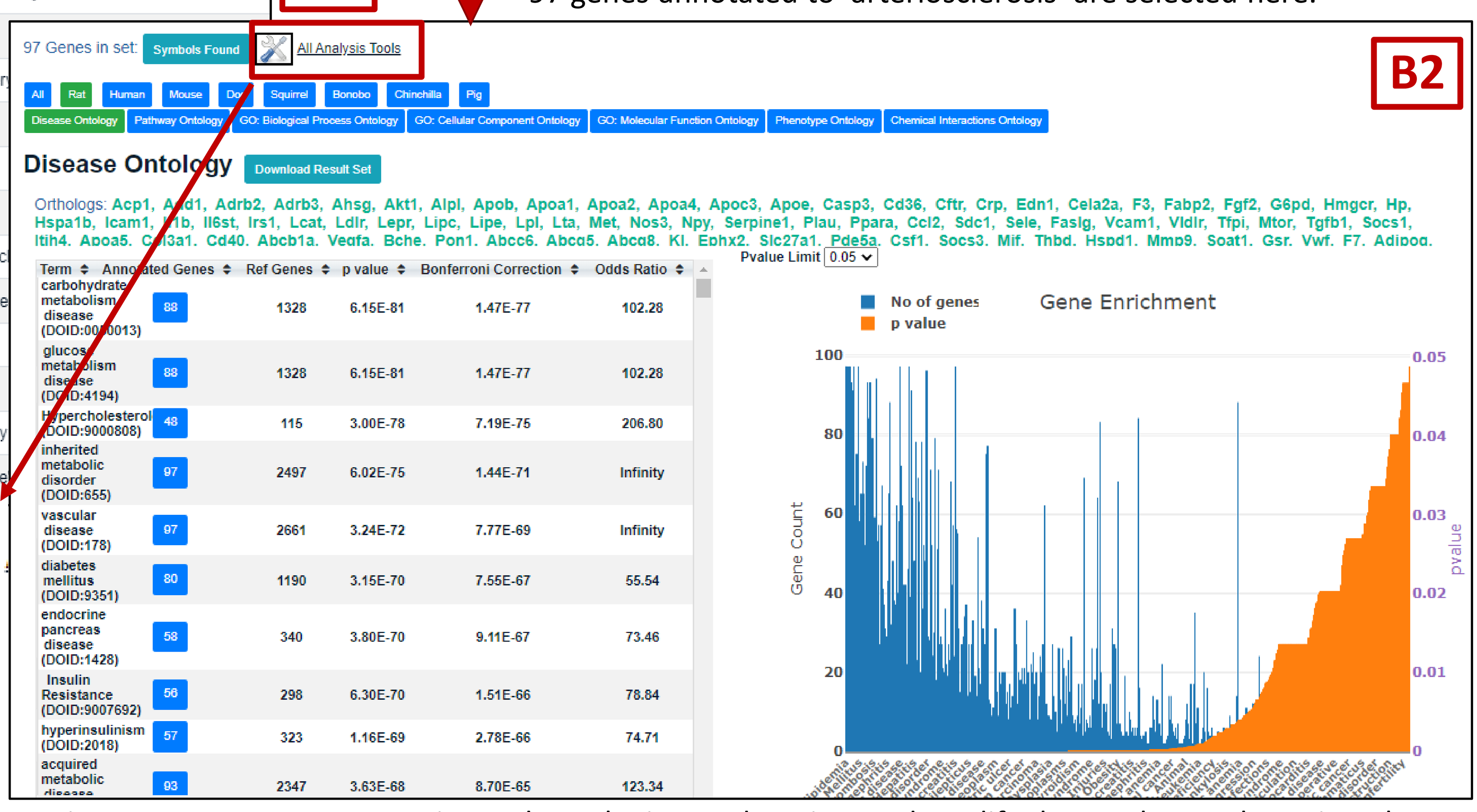

(S1B3) Use the 'All Analysis Tools' link and the tool box icon to analyze your gene list from MOET with the other RGD tools

Figure S1C: MOET provides the option to compare across species and ontologies, and to view and modify the results graph to view the results in different ways (S1C1). Click on the required species and ontology to view the list and graph of results for the selected species and ontology (S1C2). Below are the results with Human species and Pathway ontology overrepresented in the gene list entered in S1A2.

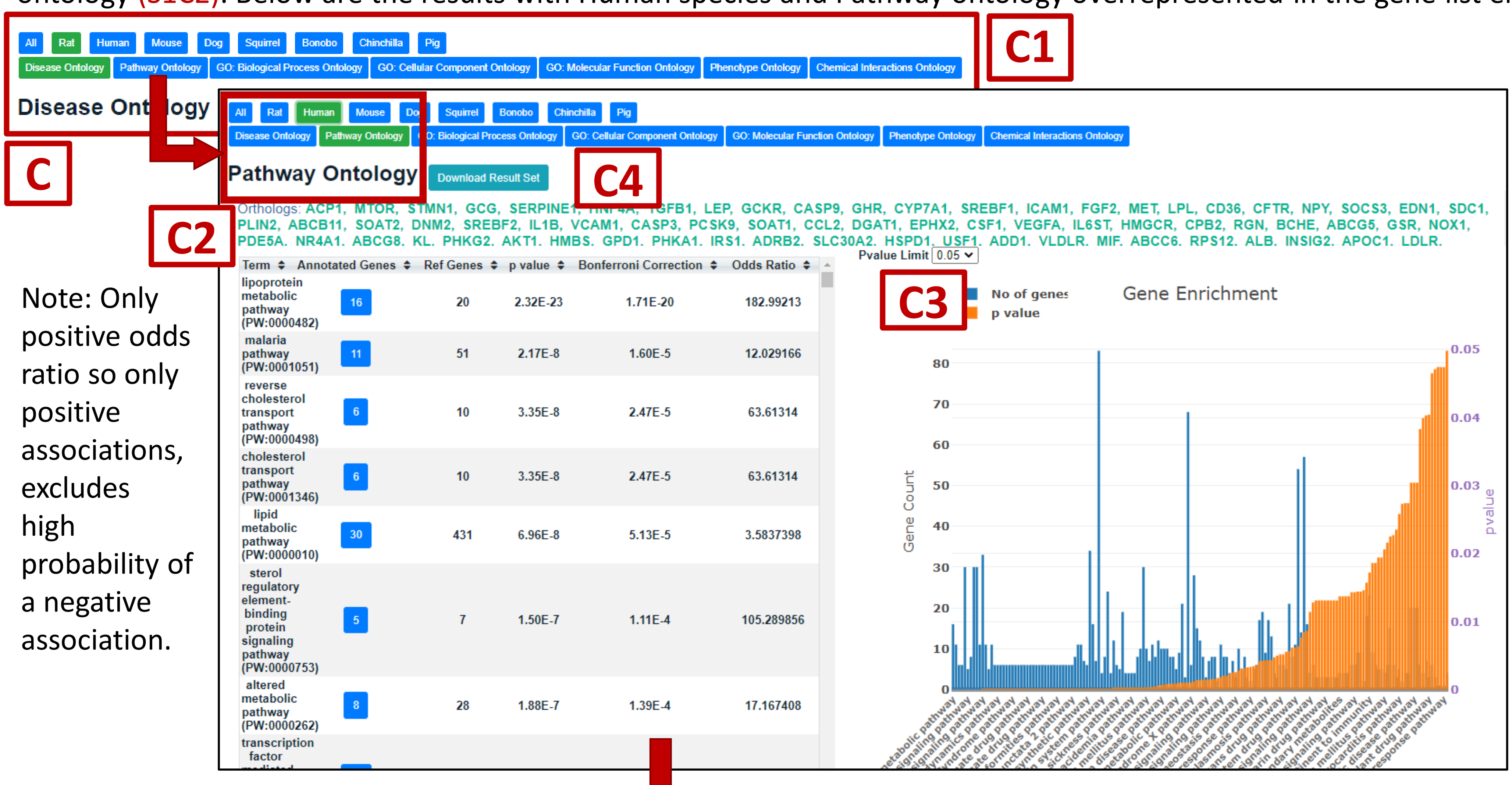

Note: Only positive odds ratio so only positive associations, excludes high probability of a negative association.

(S1C3) The default p-value limit is 0.05. You can adjust it to modify the results in the graph accordingly. (S1C3a) The 'P-value limit' drop-down shows different options to modify the P-value limit. This is the graph when the p-value was changed to 0.01. (S1C3b) Click on the 'No of genes' or 'p value' to turn off the selected option in the result output graph.

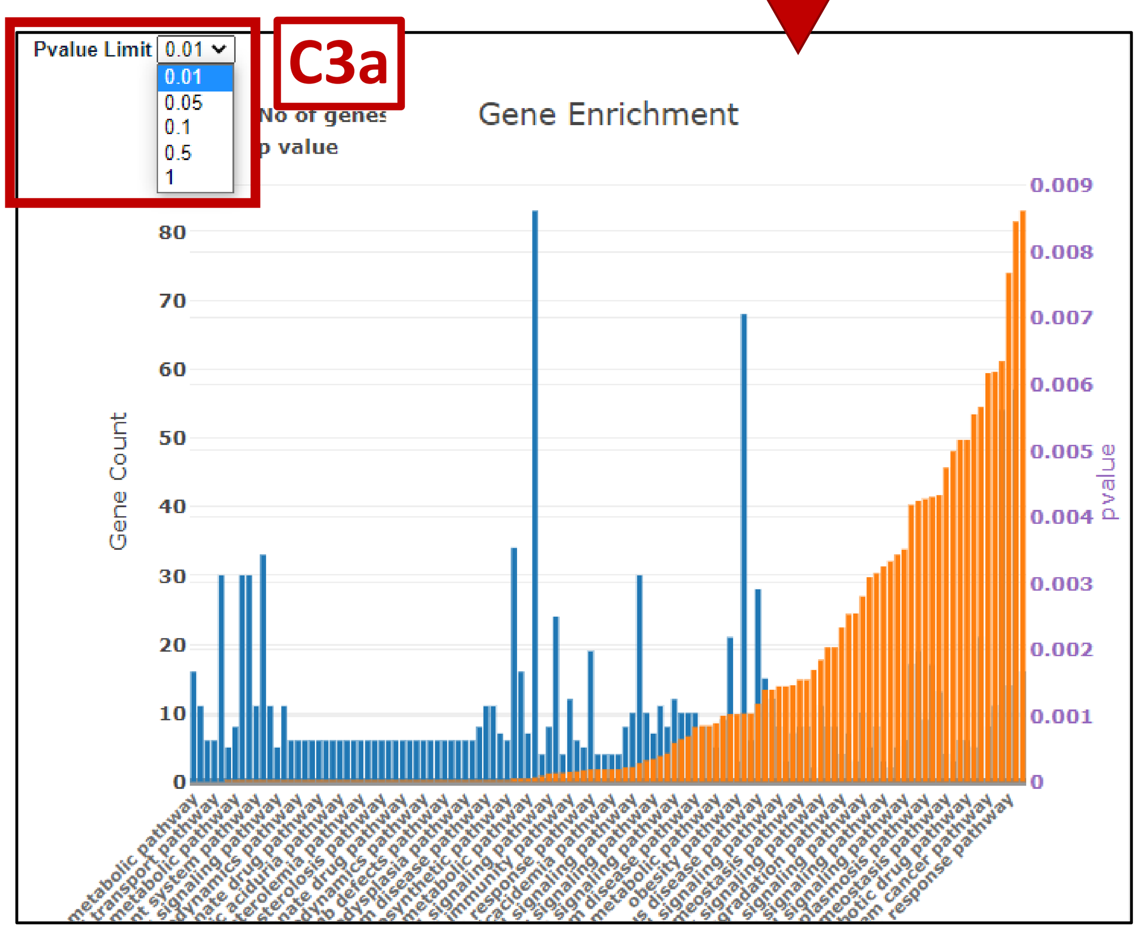

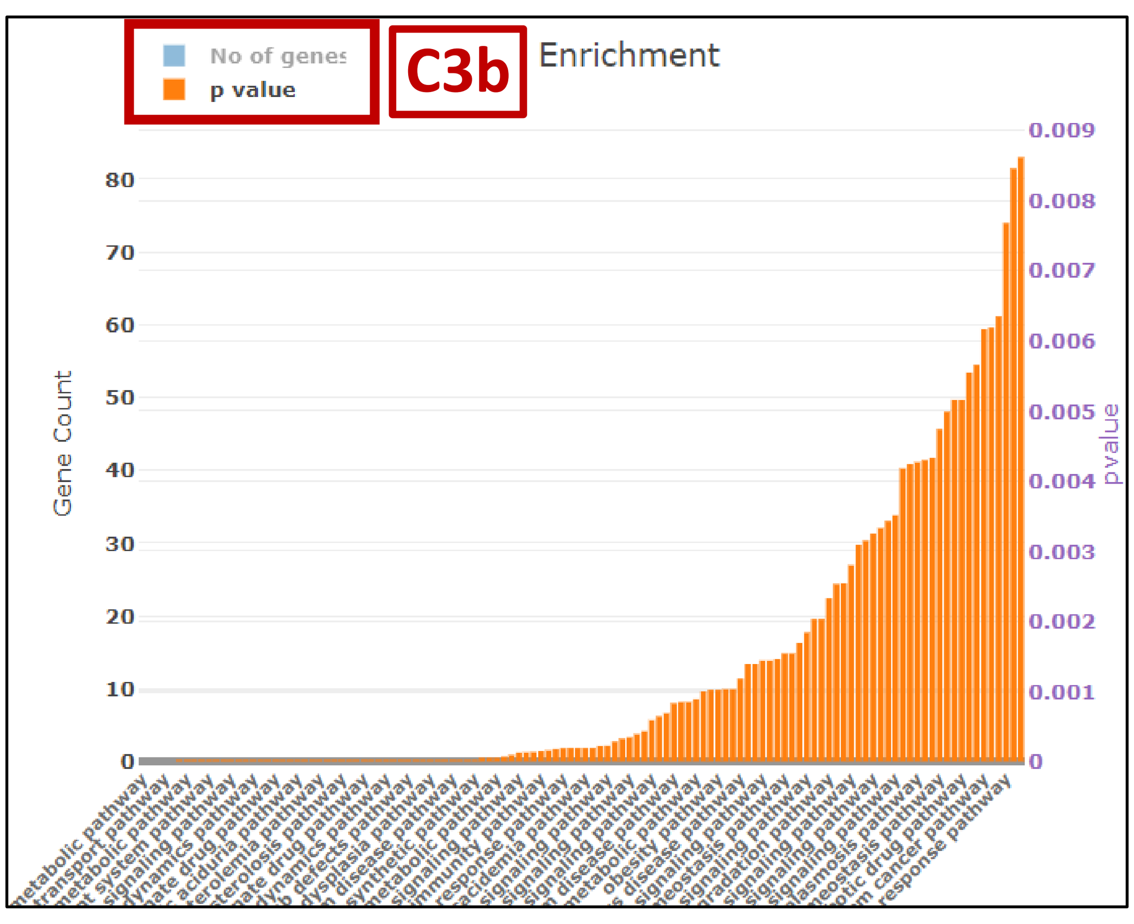

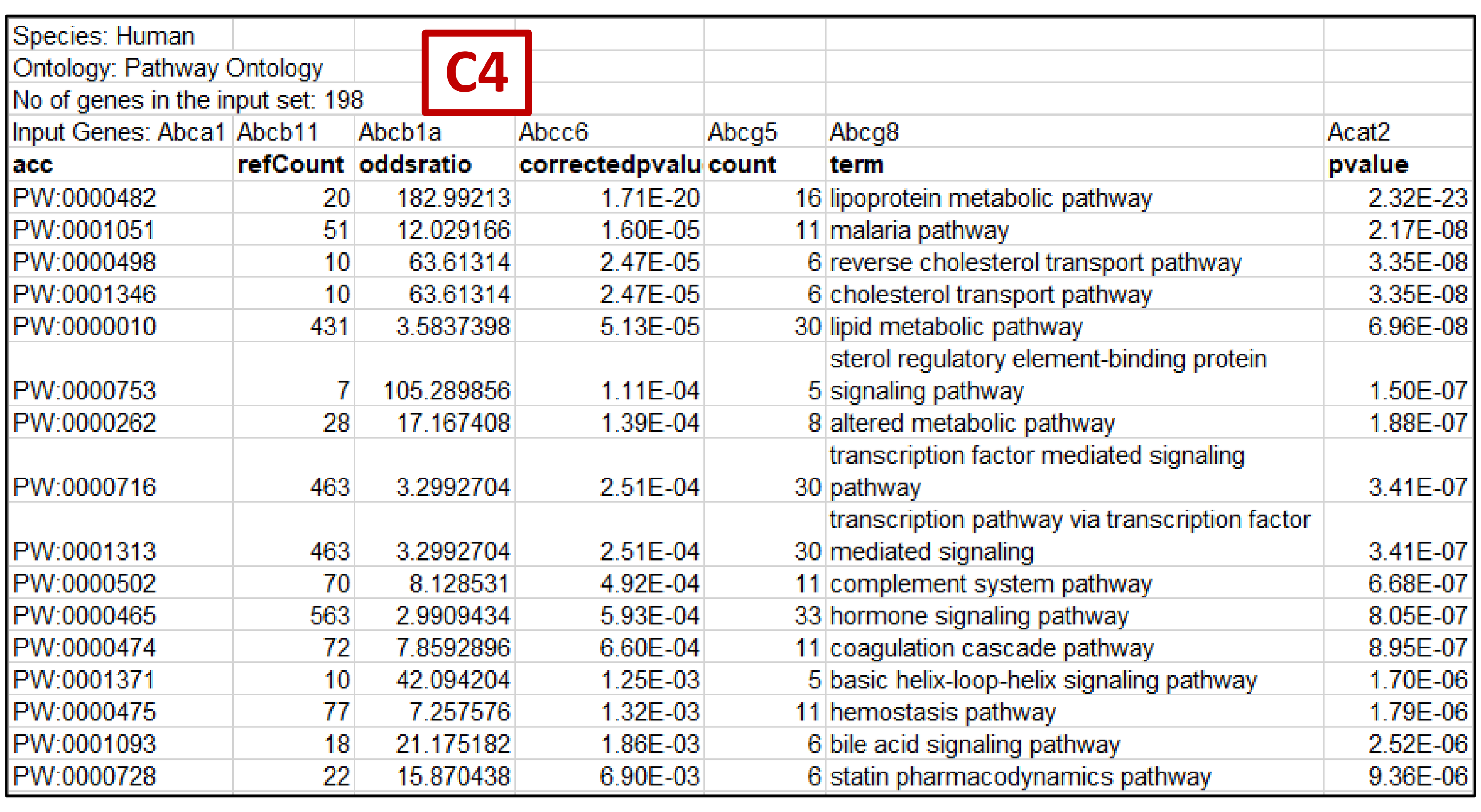

Species: Human						
Ontology: Pathway Ontology						
No of genes in the input set: 198						
Input Genes: Abca1	Abcb11	Abcb1a	Abcc6	Abcg5	Abcg8	Acat2
**acc**	**refCount**	**oddsratio**	**correctedpvalu**	**count**	**term**	**pvalue**
PW:0000482	20	182.99213	1.71E-20	16	lipoprotein metabolic pathway	2.32E-23
PW:0001051	51	12.029166	1.60E-05	11	malaria pathway	2.17E-08
PW:0000498	10	63.61314	2.47E-05	6	reverse cholesterol transport pathway	3.35E-08
PW:0001346	10	63.61314	2.47E-05	6	cholesterol transport pathway	3.35E-08
PW:0000010	431	3.5837398	5.13E-05	30	lipid metabolic pathway	6.96E-08
PW:0000753	7	105.289856	1.11E-04	5	sterol regulatory element-binding protein signaling pathway	1.50E-07
PW:0000262	28	17.167408	1.39E-04	8	altered metabolic pathway	1.88E-07
PW:0000716	463	3.2992704	2.51E-04	30	transcription factor mediated signaling pathway	3.41E-07
PW:0001313	463	3.2992704	2.51E-04	30	transcription pathway via transcription factor mediated signaling	3.41E-07
PW:0000502	70	8.128531	4.92E-04	11	complement system pathway	6.68E-07
PW:0000465	563	2.9909434	5.93E-04	33	hormone signaling pathway	8.05E-07
PW:0000474	72	7.8592896	6.60E-04	11	coagulation cascade pathway	8.95E-07
PW:0001371	10	42.094204	1.25E-03	5	basic helix-loop-helix signaling pathway	1.70E-06
PW:0000475	77	7.257576	1.32E-03	11	hemostasis pathway	1.79E-06
PW:0001093	18	21.175182	1.86E-03	6	bile acid signaling pathway	2.52E-06
PW:0000728	22	15.870438	6.90E-03	6	statin pharmacodynamics pathway	9.36E-06

(S1C4) You can click on 'Download Result Set' to download the results in the list in a .CSV format file.
