# Supplementary material for: MOET: a web-based gene set enrichment tool at the Rat Genome Database for multiontology and multispecies analyses
Source: Genetics. 2022 Jan 28;220(4):iyac005. doi: 10.1093/genetics/iyac005 (PMC8982048; doi:10.1093/genetics/iyac005)
Supplement: iyac005_Supplementary_Figure_3 [file iyac005_supplementary_figure_3.pdf]

Supplementary Figure 3

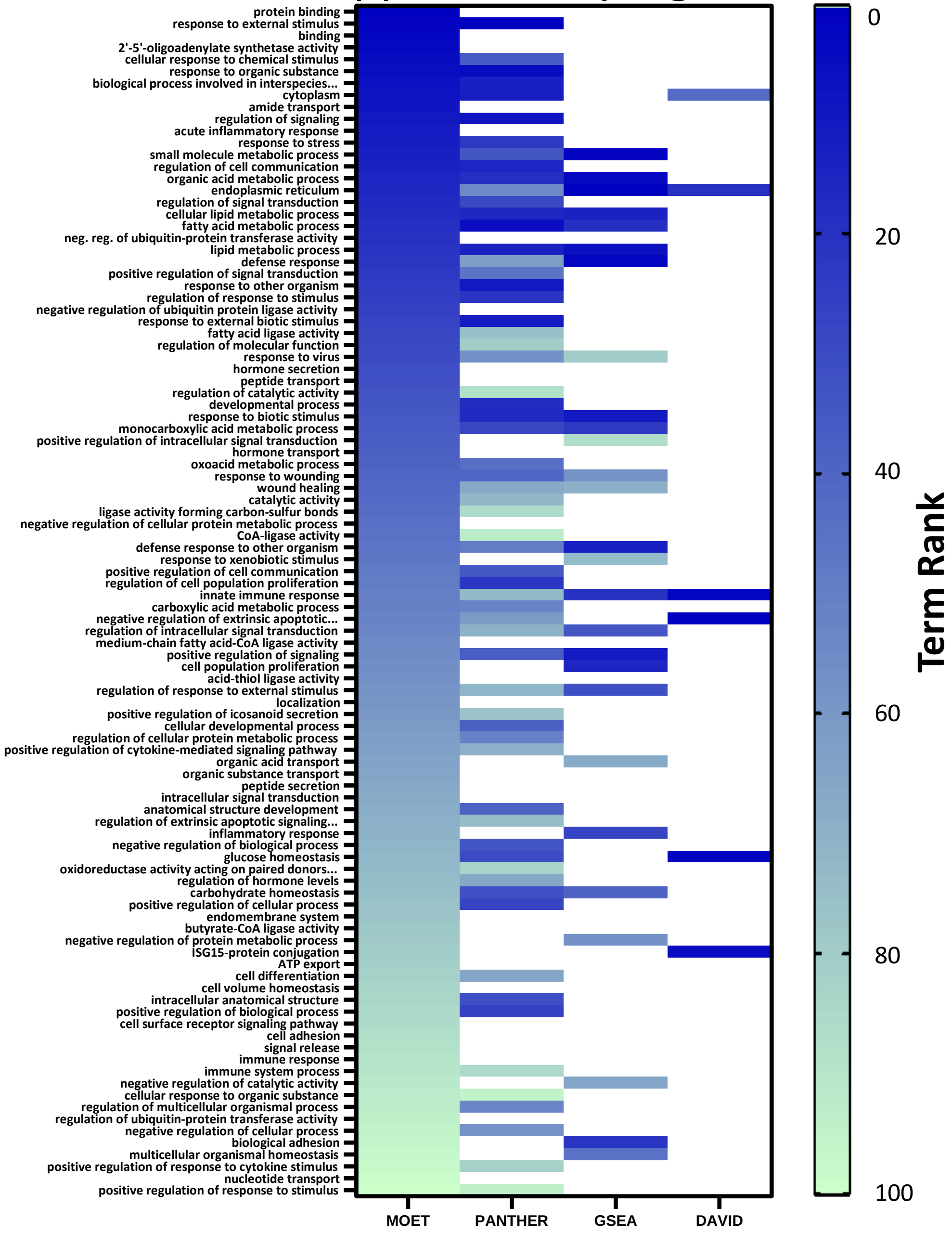

Figure S3: MOET top 100 GO term overlap with compared enrichment tools. This heat map depicts term overlap from DAVID, GSEA, and PANTHER relative to the top 100 p-value ranked GO terms from MOET. The most significant terms from each tool are shown dark blue while the less significant terms, near rank 100, are shown in green. Term names are displayed to the left of the heat map and some have been truncated due to excessive length.
