# Supplementary material for: MOET: a web-based gene set enrichment tool at the Rat Genome Database for multiontology and multispecies analyses
Source: Genetics. 2022 Jan 28;220(4):iyac005. doi: 10.1093/genetics/iyac005 (PMC8982048; doi:10.1093/genetics/iyac005)
Supplement: iyac005_Supplementary_Figure_2 [file iyac005_supplementary_figure_2.pdf]

Supplementary Figure 2

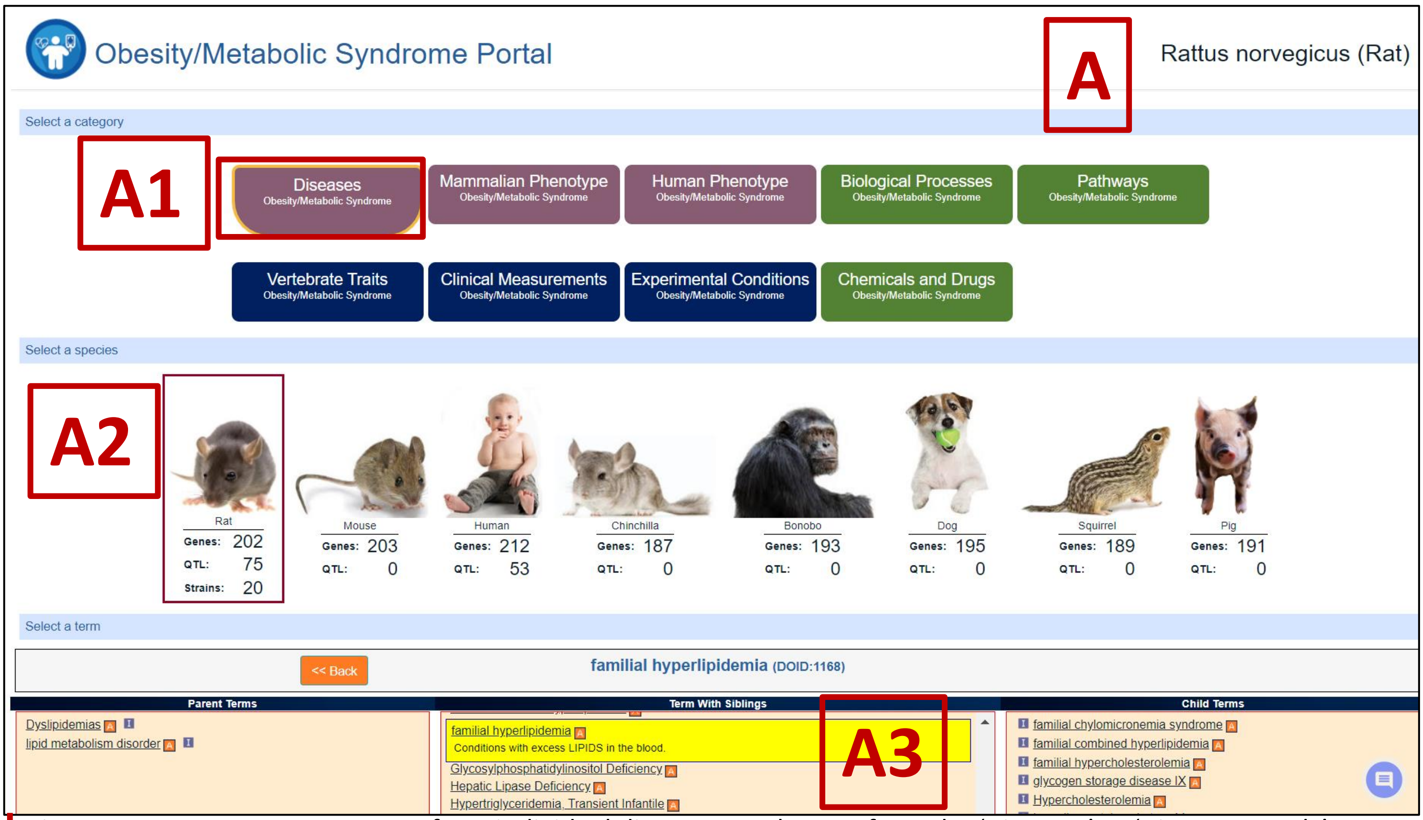

Figure S2A: You can access MOET from individual disease portal pages from the 'Diseases' or 'Disease Portals' menu on the RGD front page. (S2A1) Default selection is 'Diseases' but you can select other ontologies by clicking on them. (S2A2) Similarly, other species can be selected by a single click. (S2A3) You can find the disease-related term from the term browser with its definition below it.

Figure S2B: The number of genes, QTL and strains annotated to your selected term are displayed and can be downloaded.

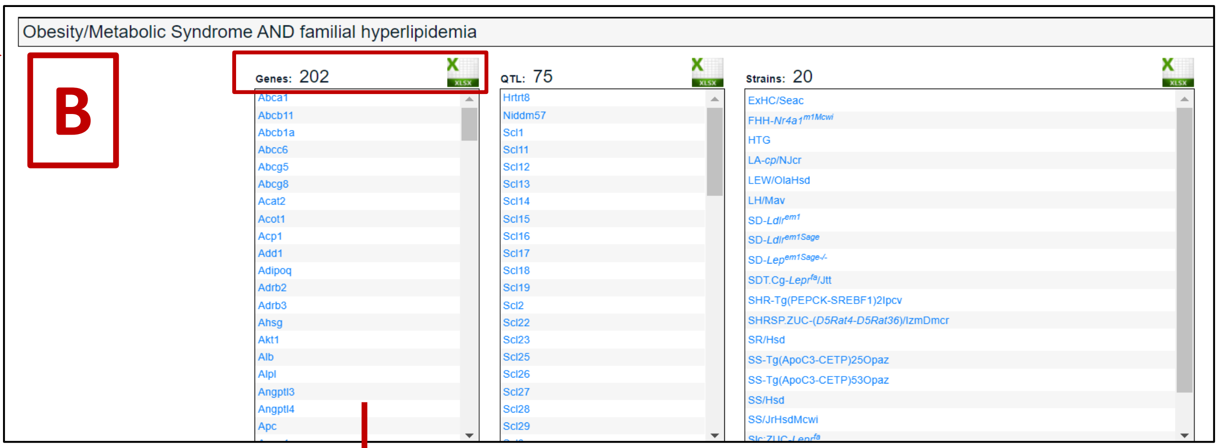

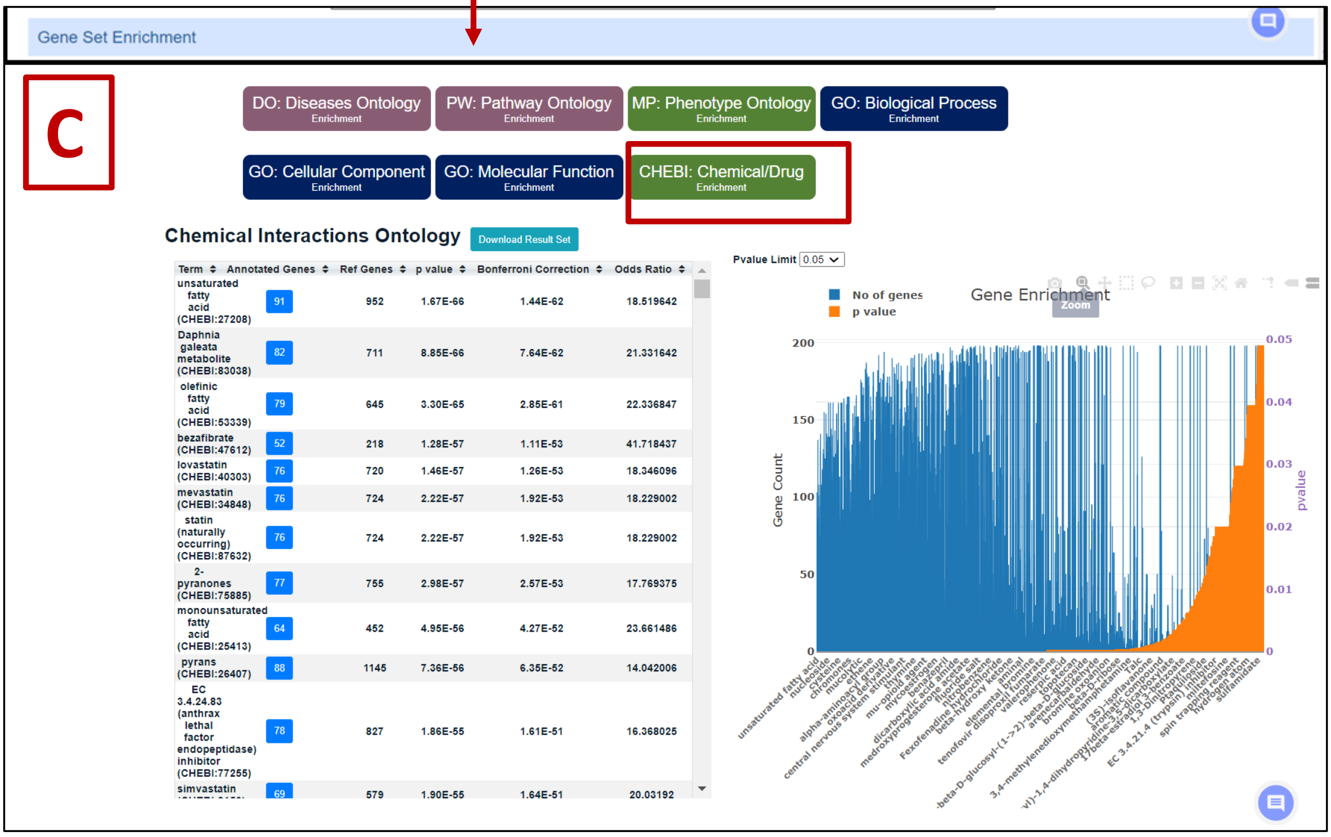

Figure S2C: MOET is integrated with individual disease portal pages and is at the bottom on individual pages. You can interchangeably perform MOET analysis for different ontologies that you selected from the ontology tabs below the 'Gene Set Enrichment' option, annotated to the list of input genes. The results shown here are for the list of ChEBI ontology terms annotated to the input gene list. You can select other ontologies or species from the top of the same page as well.
